# Retest Reliability of Individual P3 Topography Assessed by High Density Electroencephalography

**DOI:** 10.1371/journal.pone.0062523

**Published:** 2013-05-01

**Authors:** Manuel Vázquez-Marrufo, Javier J. González-Rosa, Alejandro Galvao-Carmona, Antonio Hidalgo-Muñoz, Mónica Borges, Juan Luis Ruiz Peña, Guillermo Izquierdo

**Affiliations:** 1 Experimental Psychology Department, Faculty of Psychology, University of Seville, Seville, Spain; 2 Multiple Sclerosis Unit, Virgen Macarena Hospital, Seville, Spain; 3 Laboratory for Clinical Neuroscience, Centre of Biomedical Technology (CTB), Technical University of Madrid (UPM), Madrid, Spain; Institute of Psychology, Chinese Academy of Sciences, China

## Abstract

**Background:**

Some controversy remains about the potential applicability of cognitive potentials for evaluating the cerebral activity associated with cognitive capacity. A fundamental requirement is that these neurophysiological parameters show a high level of stability over time. Previous studies have shown that the reliability of diverse parameters of the P3 component (latency and amplitude) ranges between moderate and high. However, few studies have paid attention to the retest reliability of the P3 topography in groups or individuals. Considering that changes in P3 topography have been related to different pathologies and healthy aging, the main objective of this article was to evaluate in a longitudinal study (two sessions) the reliability of P3 topography in a group and at the individual level.

**Results:**

The correlation between sessions for P3 topography in the grand average of groups was high (r = 0.977, p<0.001). The within-subject correlation values ranged from 0.626 to 0.981 (mean: 0.888). In the between-subjects topography comparisons, the correlation was always lower for comparisons between different subjects than for within-subjects correlations in the first session but not in the second session.

**Conclusions:**

The present study shows that P3 topography is highly reliable for group analysis (comprising the same subjects) in different sessions. The results also confirmed that retest reliability for individual P3 maps is suitable for follow-up studies for a particular subject. Moreover, P3 topography appears to be a specific marker considering that the between-subjects correlations were lower than the within-subject correlations. However, P3 topography appears more similar between subjects in the second session, demonstrating that is modulated by experience. Possible clinical applications of all these results are discussed.

## Introduction

The study of human cognition is one of the biggest challenges in neuroscience. One crucial aspect is to obtain measures that allow it to be studied objectively. It is desirable that such measures be stable over time while the cognitive mechanism is engaged in performing the task. For several decades, multiple studies have been conducted to check the stabilities of different measures of cerebral activity based upon electroencephalography (EEG) and more specifically in the cognitive potentials field (for instance, the P3 component). It is necessary at this point to emphasize that when studying the retest reliability of an ERP there are two factors at play: a) the measurement of the signal may be noisy (for example due to recording artifacts) and b) the signal itself that may vary from one session to the next. Therefore, the identification of traits in this kind of analysis must be cautious.

To study the reliability of the P3 parameters, diverse issues have been considered in the design of these tests: (a) which parameters are to be analyzed in the study (latency, amplitude and/or topography); (b) whether the subjects in a group are to be compared with each other or with a comparative group; (c) what cognitive paradigm is to be used (oddball, stroop, etc); (d) whether stability is to be studied in a single session (for example, comparing the first and second halves of the experiment) or whether time should elapse between the repeated measures (days, weeks, months, years). Since the present study is focused on stability among sessions separated by intervals typically employed in longitudinal studies (pharmacological treatments, neuropsychological rehabilitation programs, etc.), no review of studies focused on intrasession stability will be included (detailed information can be found in [1], [2], [3]).

The results of studies to check stability between two sessions separated by periods of days, months or even years have commonly suggested that the P3 parameters (latency and amplitude) show a moderate to high level of reliability (ranging from 0.40 to 0.99) (see [1], [4], [5], [6]). However, most of these studies analyzed a small number of electrodes and could not examine the reliability of the P3 topography in follow-up studies with high density EEG. One of the studies in this direction [3], using waveform cross-correlation coefficients, showed that the map of the P3 component was very stable for intersession periods from 15 minutes to one month. Fallgater et al.,[7], using a go/no go paradigm, demonstrated that reliability of P3 topography was very high (Pearson r value >0.85) and suggested that this type of analysis can be used as electrophysiological trait markers of the human brain. More recently, Gruendler et al. [8] investigated reliability in a lateralized time-estimation task. Using the Global Map Dissimilarity Index, they found a large topographical overlap for all components (N2, error related negativity (ERN), and feedback related negativity (FRN)) analyzed in the study (≥0.85).

Another question arises when the diverse P3 parameters are compared among different groups of subjects. This is a crucial aspect in clinical studies where pathological and control groups are compared. In these cases, various studies have shown that the reliability of both P3 parameters (amplitude and latency) is low. For example, Dustman and Beck [9] observed considerable differences among the participants in cognitive potentials during the first 300 ms and they concluded that the response to visual stimuli is highly specific in human subjects. Later, in 1965, the same authors [10] studied the correlation between twins (identical and non-identical) and found a bigger r value for the identical (0.81) than the non-identical (0.54). This latter score was close to the value found for unrelated children matched for age (0.56). These results suggest that the definition of cognitive potentials is specific for each human subject and genetic factors are determinants of them. Zamrini et al., [11], in a study of intersession stability (one month) using an auditory oddball paradigm, found high variability among subjects for the P3 parameters (latency and amplitude), although no differences were found when the mean of all subjects was used. However, another study that checked the reliability of P3 topography in six different laboratories [12] showed that the scalp distribution yielded good to excellent agreement across laboratories with different subjects.

The general conclusion from the literature cited above is that the P3 topography is highly stable when grand averages of one group (comprising the same subjects) are compared. However, when the comparison between topographical maps includes different samples of subjects, lower correlations are found. All these analyses have been conducted on groups, as appropriate for global studies of the changes induced by rehabilitation programs or drug therapies. However, in the clinical context, one of the main purposes is to evaluate simple cases, not including the patient in a group but assessing his/her status or evolution independently. Therefore, the main aim of the present study is to perform within- and between-subjects comparisons for the P3 topography of individual subjects.

### Predictions

The first result expected is to support previous investigations in which behavioural and P3 latency and amplitude show high reliability for the oddball paradigm. Another prediction is that the reliability of P3 topography when grand averages of a group are compared will be high as evidenced by previous studies. In particular, the use of a simple cognitive task (visual oddball) will produce better reliability than in other studies with supposedly more complex cognitive setups. A third purpose of this study is to determine whether the individual P3 topography is highly specific for each subject in two sessions. We predict a specific topography for each subject, though common features among the subjects are also evidenced when groups are analyzed. Lastly, retest reliability for the individual P3 topography between different subjects will be analyzed to determine whether the experimental procedure could make them more similar with repetition of the task. An affirmative result will reveal that the individual P3 topography can be modulated by experience and not determined only by the genetics of the subjects.

## Materials and Methods

### Ethics Statement

This study was carried out in compliance with the Helsinki Declaration. All participants signed informed consents before their inclusion and the protocol was approved by the ethics committee of the University of Seville (project code: SEJ2007-65343).

### Participants

Thirty adults were recruited from university students and staff. No formal neurological evaluation of the subjects was performed, but all were in good health and without significant neurological history or any kind of drug consumption. All subjects participated in two EEG recordings separated by a mean of 48.5±47.1 days (range from one week to three months). After completion of the recording phase, eight subjects were rejected owing to artifacts impossible to remove in at least one electrode of the 52 montage. No interpolation procedures were applied, to preclude modifications of the data that could affect further topographical analysis. The final sample was composed of 22 adults (8 males, 14 females) ranging in age from 21 to 50 years (mean 28.3±7.68 years (all but one were right-handed). In view of previous reports about differences in P3 parameters (latency and amplitude) being related to some biological determinants [13], the time at which recording was started, sleeping hours, and ingestion of caffeine and nicotine were matched among sessions.

### Cognitive Task

The paradigm employed in the present study was a “visual oddball” that consisted in the discrimination of uncommon visual stimuli in a sequence of frequent stimuli. The target stimulus (appearance probability: 25%) was a rectangle with a checkerboard pattern comprising red and white squares. The standard (frequent) stimulus was equivalent in size with the same pattern but with black and white squares. Both stimuli were presented in the same position in the centre of the screen. A fixation point was present when no stimuli were displayed to prevent changes in eye position during the experiment. The screen was located 70 centimetres from the participant's eyes and the size of both stimuli was 7.98 of visual angle on the X axis and of 9.42 on the Y axis. Both stimuli were presented for 500 milliseconds (ms) and the interstimulus interval was one second, during which the subject could respond. One block with 200 trials was used in a pseudorandom presentation. The task for the participants was to press the mouse button with the right index finger when a target stimulus appeared but to ignore the standard stimulus. At the end of the experimental session, reaction time and percentage accuracy (for the target and overall, including no responses for the standard stimuli) were calculated.

### EEG Procedure

**Figure 1:**
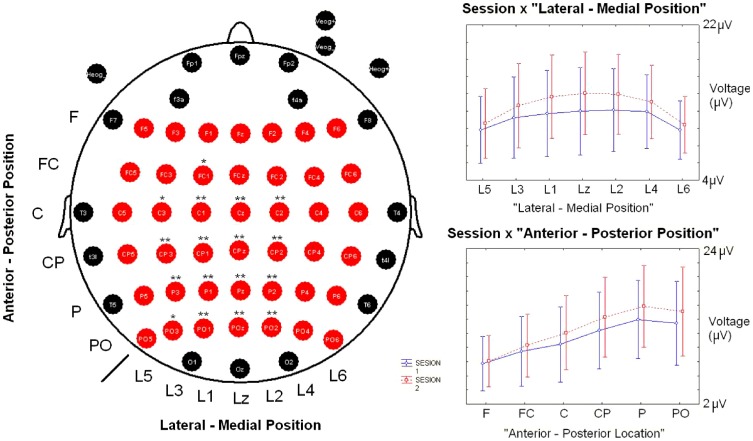
Electrode array and statistically significant interactions in the ANOVA of the amplitude. On the left side, fifty-eight of the 64 EEG electrodes used are depicted. The red electrodes were used to analyze the amplitude differences between sessions. Statistical results for the ANOVA (after Bonferroni correction) in the comparison of amplitudes between sessions are coded as * p<0.05 and ** p<0.001. On the right side, graphics for interactions that were statistically significant in the ANOVA are displayed. Abbreviations: F (frontal), FC (Frontocentral), C (Central), CP (Centroparietal), P (Parietal), PO (Parietooccipital). L (line), z (zero or midline).

The electroencephalogram was recorded from 58 scalp electrodes (Fp1, Fpz, Fp2, AF3, AF4, F7, F5, F3, F1, Fz, F2, F4, F6, F8, FC5, FC3, FC1, FCz, FC2, FC4, FC6, T3, C5, C3, C1, Cz, C2, C4, C6, T4, TP7, CP5, CP3, CP1, CPz, CP2, CP4, CP6, TP8, T5, P5, P3, P1, Pz, P2, P4, P6, T6, PO7, PO3, PO1, POz, PO2, PO4, PO8, O1, Oz, O2; see figure 1 for detailed locations of recording derivations). All electrodes were referenced during the recording to the linked earlobe channel and offline re-referenced to an averaged reference. The ground electrode was placed in the mid-forehead. Vertical and horizontal electrooculograms (VEOG and HEOG) were recorded with bipolar recordings from electrodes situated in the inferior and superior positions of the left orbit and in the external canthi of the ocular orbits, respectively. The electrode signals were amplified using BrainAmp amplifiers (Brain Products GmbH, Germany) and digitally stored using Brain Vision Recorder software (Brain Products GmbH, Germany). The EEG signal was digitized at a frequency of 500 Hz, filtered in the amplifier using a band-pass of 0.01–100 Hz with the impedance below 5 kOhm during the experiment. The following protocol was applied to calculate the cognitive potentials: ocular correction of the blinking artefact in the scalp electrodes using the algorithm developed by Gratton et al., [14]; segmentation of the continuous EEG recording (–100 to 1000 ms, zero being the onset of the target stimulus); baseline correction based on the previous interval to the stimulus (–100 to 0 ms); visual review of EEG epochs and rejection of artefacts. Also, trials in which the HEOG signal was outside the ±75 µV range were rejected. Lastly, averages were calculated for the target stimulus and for each subject. As recommended by Polich [15], all the individual averages comprised at least 20 artifact-free trials (session 1: 46.7; session 2: 47.7). The latency and amplitude values of the P3 component were calculated in the electrode that showed the maximum amplitude for each subject. The P3 component was identified as the maximum positivity in the interval between 300 and 450 milliseconds. For better determination of the peak, a low pass filter (30 Hz (48dB/octave)) was used to eliminate small high-frequency fluctuations. After the latency was determined by the maximum amplitude, amplitude values for the rest of the electrodes were exported in the same latency for topographical study, as some authors suggest [16].

### Data Analysis

The statistical method used to calculate possible differences among sessions in behavioural responses and in the latency and amplitude of the P3 component was a paired t-test for dependent variables. For the study of topographical differences in the amplitude of the P3 component between the two sessions, an analysis of variance (ANOVA) was applied with the following factors: Factor 1: “Session” (levels (2): 1 and 2); Factor 2: “Antero-posterior Position” of the electrode (levels (6): Frontal; Frontocentral; Central; Centroparietal; Parietal; Parietooccipital); Factor 3: “Lateral-Medial Position” (levels (7): from lateral left to lateral right, example: Line 5, Line 3, Line 1, Midline or Line zero (z), Line 2, Line 4, Line 6) (i.e. F5, F3, F1, Fz, F2, F4, F6)(see figure 1 for the locations of the electrodes analyzed). All variables were checked for normality using the Shapiro-Wilk test. A Greenhouse-Geisser correction for sphericity was applied. A Bonferroni correction was carried out in multiple comparisons post-hoc analysis. In all these analyses, a probability of p<0.05 was considered significant.

To analyze the correlations between the amplitudes of the P3 component in two sessions and within-subjects, the intraclass correlation test (ICC) was used. Pearson’s product-moment r was employed for the between-subject comparisons. As suggested by Kileny and Kripal [17], the 0.05 significance level was divided by the number of contrasts made for both correlation analyses (within- and between-subjects). For the within-subjects comparison, the new level of significance obtained was p = 0.002 (0.05 divided by 22 comparisons) and for the between-subjects correlation, the p value was established as <0.00001 (0.05/462 comparisons). Lastly, the coefficient of variation (CV) was calculated for all parameters using the formula described by other authors [18] (Coefficient of Variation  =  (Standard Deviation/Mean)×100).

## Results

### Behavioural Data

The reaction times did not differ significantly between the two sessions (session 1: 314±32; session 2: 312±34 ms) (t = 1.10, p = 0.280) (see table 1 for individual values of all the behavioural and ERP parameters). Nor were significant differences found in the percentage accuracy of global performance (target and standards) (session 1: 97.5±4.5; session 2: 97.3±4.7) (t = 0.302, p = 0.765) or the specific percentage accuracy for the target stimulus (session 1: 99.2±1.2; session 2: 99.1±1.2) (t = 0.641, p = 0.528). On the other hand, all these variables showed highly significant correlations between the two sessions (RT r = 0.880, p<0.001; global accuracy r = 0.860, p<0.001; target accuracy r = 0.827, p<0.001). Table 1 gives the values in sessions 1 and 2 for all the parameters analyzed in the present study for each subject. The table demonstrates that some subjects showed an increase in reaction time between sessions (maximum 29 ms) and others a decrease (maximum 24 ms). Regarding accuracy, changes were minimal for both target and global score.

**Table 1:** Behavioral and ERP parameters for each subject.

Subject	RT S1	RT S2	RT Dif	ACC T S1	ACC T S2	ACC T Dif	ACC G S1	ACC G S2	ACC G Dif	Lat S1	Lat S2	LatDif	Amp S1	Amp S2	AmpDif	Elect S1	Elect S2
1	408	406	2	94.5	94.5	0	80	78	2	388	398	–10	11.79	13.6	–1.81	P3	Pz
2	279	279	0	100	100	0	100	100	0	306	310	–4	21.21	21.42	–0.21	Pz	Pz
3	308	284	24	100	100	0	100	100	0	348	350	–2	14.95	18.93	–3.98	PO1	PO1
4	324	315	9	99	99.5	–0.5	96	100	–4	336	348	–12	11.43	15.3	–3.87	P3	Pz
5	343	337	6	98	98	0	92	96	–4	364	330	34	6.8	8.03	–1.23	T5	Pz
6	302	287	15	99.5	100	–0.5	98	100	–2	346	350	–4	9.55	14.5	–4.95	PO3	POz
7	314	324	–10	99.5	100	–0.5	98	100	–2	356	334	22	16.16	19.05	–2.89	POz	P3
8	318	294	24	100	100	0	100	100	0	356	346	10	11.25	12.02	–0.77	P4	CP2
9	351	372	–21	99	98	1	98	92	6	396	410	–14	10.59	14.39	–3.8	P3	CP3
10	270	253	17	100	99.5	0.5	100	100	0	352	350	2	15.22	14.91	0.31	P6	P4
11	295	324	–29	100	100	0	100	100	0	332	352	–20	17.14	17.78	–0.64	PO2	PZ
12	284	285	–1	100	99.5	0.5	100	98	2	340	342	–2	15.92	16.88	–0.96	PO5	PO3
13	333	318	15	100	99	1	100	96	4	422	368	54	13.7	14.78	–1.08	P2	Pz
14	296	308	–12	99.5	100	–0.5	100	100	0	374	336	38	13.98	17.45	–3.47	P3	PO1
15	310	325	–15	100	99	1	100	96	4	328	336	–8	13.4	12.08	1.32	Pz	Pz
16	315	307	8	100	99.5	0.5	100	98	2	320	344	–24	18.03	19.82	–1.79	Pz	CPz
17	342	326	16	98.5	99	–0.5	94	96	–2	364	380	–16	15.46	17.05	–1.59	CP2	Pz
18	259	262	–3	100	100	0	100	100	0	322	322	0	15.26	14.73	0.53	POz	PO4
19	300	319	–19	99	98	1	98	96	2	304	328	–24	12.55	11.86	0.69	CP2	Cz
20	300	293	7	99.5	100	–0.5	100	100	0	358	354	4	13.37	14.34	–0.97	POz	PO5
21	351	326	25	98	99.5	–1.5	92	98	–6	330	336	–6	13.23	13.62	–0.39	T5	PO1
22	312	316	–4	99.5	98.5	1	98	96	2	328	332	–4	21.09	24.09	–3	PO3	PO1
**Mean**	**314**	**312**		**99.2**	**99.1**		**97.5**	**97.3**		**349**	**348**		**14.18**	**15.70**			
**StdDev**	**32**	**34**		**1.2**	**1.2**		**4.5**	**4.7**		**29**	**24**		**3.4**	**3.5**			
**CV**	**10.3**	**10.8**		**1.3**	**1.3**		**4.8**	**5.0**		**8.3**	**6.8**		**24.2**	**22.7**			

Abbreviations. RT: Reaction time (in milliseconds). S1: Session 1. S2: Session 2. ACC: Accuracy. T: Target. G. Global (Target and Standard). Dif. Difference (S1 – S2). Lat. Latency (in milliseconds). Amp. Amplitude (in microvolts). Elect (electrode with the maximum amplitude value for P3). CV: Coefficient of Variation.

### P3 Latency and Amplitude

**Figure 2:**
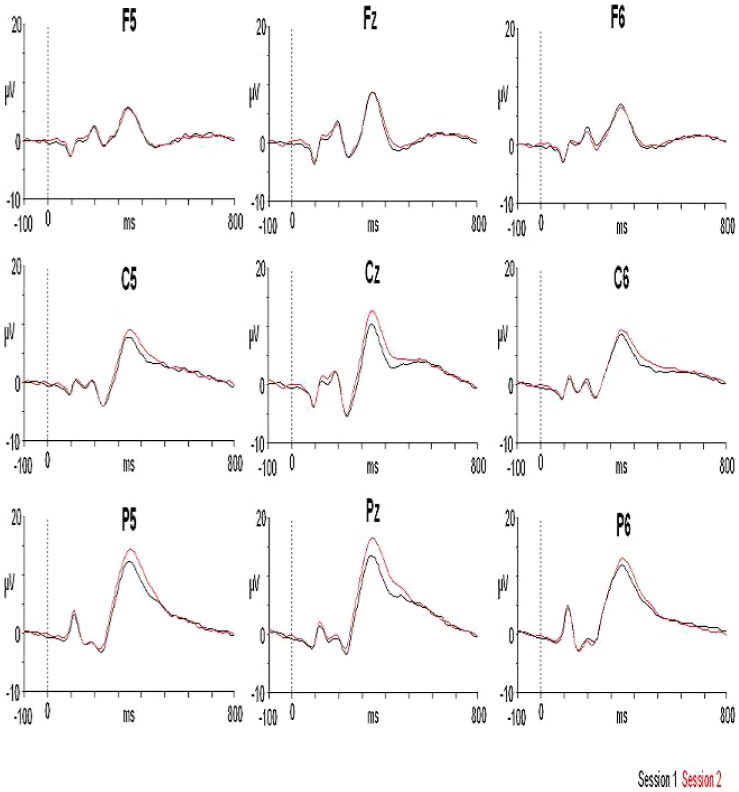
Grand average ERPs for both sessions in nine selected electrodes. The X axis represents “time” expressed in milliseconds (ms) and the Y axis the “amplitude” of the ERP in microvolts (µV). The vertical dashed line indicates the onset of the stimulus. The black trace is for session 1 and the red trace for session 2. Note the increase in the P3 amplitude especially in centro-parietal derivations.

In the latency analysis, no intersession differences were found between the two measures (session 1: 349±29 ms; session 2: 348±24 ms) (t = 0.274, p = 0.787). As for the behavioural variables, the latency showed a high correlation between the two sessions (r: 0.723, p<0.001). For amplitude, there was a statistically significant increase in the second session (session 1: 14.18±3.4 µV; session 2: 15.7±3.5 µV) (t = –4.279, p<0.001) (see figure 2). The correlation between the two variables was even higher than for the latency (r: 0.880). The P3 latency showed changes reaching 54 ms in one case, though for almost half the sample (11 subjects) the difference between sessions did not exceed 10 ms. The amplitude exhibited a change of 4.95 microvolts in one subject, and as general rule (occurred in 18 subjects) an increment was found between sessions.Finally, the coefficient of variation for all the parameters (behavioural, latency and amplitude of the P3 component) showed very acceptable values (see table 1 for the different values for each parameter). In particular, the smallest coefficient of variation was obtained in the accuracy for the responses to the target stimulus (CV session 1: 1.3; session 2: 1.3) and more variation was obtained for the amplitude of the P3 component (CV. Session 1: 24.2; session 2: 22.7).

### P3 Topography (Group Analysis)

In the analysis of modulations through the scalp, the ANOVA showed different interactions between factors (Session x Antero-posterior position: F(5,29.7) = 4.07, p = 0.002 and Session x Lateral-Central position: F(6,23.5) = 6.86, p<0.001). Posthoc analysis showed that these interactions were caused by statistically significant differences in some of the electrodes from the scalp concentrated around the Pz electrode (see figure 1 for complete list of these electrodes and their p-corrected values). Both grand averages exhibited a really high correlation between the two sessions (r = 0.977, p<0.001).

### P3 Topography (Individual Analysis, Within-Subjects)

**Figure 3:**
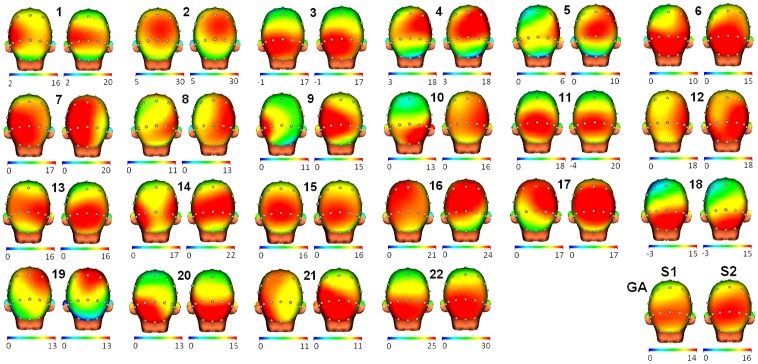
3D head maps for each subject in both sessions. Pairs of 3D head maps are displayed for each of the 22 subjects participating in the experiment and the grand average (GA). The left side of the pair is the P3 topography in session 1 and the right is for session 2. Note that the scale (in microvolts) has been adjusted for each subject between session 1 and 2 to show clearly the general increase in P3 amplitude for session 2 and the similar topography among sessions.

In the scalp distribution analysis, the first obvious result was the discrepancy in the electrode that presents the maximum voltage for the P3 component in both sessions and for every subject (see table 1). Remarkably, this difference in location was in some cases a subtle shift to a close electrode.When the correlations in topography for each individual participant were analyzed (intraclass correlation, ICC), the results showed that practically all subjects were very similar in the two sessions (r between 0.626 and 0.981, average: 0.888) (see figure 3 and table S1). In all cases, the level of significance of these correlations was p<0.001, which guarantees that they are within the significance bounds despite the multiple comparisons.

### P3 Topography (Individual Analysis, Between-Subjects)

When different subjects were compared, the correlation levels were generally below the within-subject value. The only exception in the first session was subject 21, who correlated more strongly (0.750) with participant 9 than with himself (0.683). If this analysis were carried out in the second session, diverse comparisons showed higher correlations in the between-subject than the within-subject comparisons. For instance, subject number three showed a high ICC (0.934) and no subject from the sample had a correlation score over 0.92. However, in the second session, three subjects (6,11 and 22) showed correlations even higher than 0.934 (r: 0.94, 0.96 and 0.97) (see table S1 for specific values of all these comparisons).

## Discussion

### Behavioural Data

The behavioural responses exhibited a high reliability level when group analysis was performed, as described in other studies [6]. In the analysis of individual measures, the descriptive study of variations in latency and amplitude showed considerable changes in some subjects, but not in others, where the variation was very small. However, it is notable that when the variations were wider, the coefficients of variation were similar to those in some clinical tests (CV under 30) [18]. This result has been obtained by other authors [12] and supports the idea that the behavioural responses are highly stable, like other clinical parameters.

### P3 Latency and Amplitude

Latency as much as amplitude showed high correlations between the two sessions, as reported by other authors [3], [4], [5], [6], [9], [11], with high levels for both measures (r = 0.723 for latency and r = 0.880 for amplitude). In addition, the correlation was higher for amplitude than for latency, as has been pointed out in several studies [2], [5], [6], [19]. There was no statistically significant change in latency between the two sessions but the amplitude increased in the second session, as described in other studies [6], [20]. These results reveal that notwithstanding a high correlation between sessions, the amplitude could show an overall change. This is a particularly relevant result since some studies have reported increases in amplitude in pathological samples, which can be interpreted as a consequence of the application of certain treatments. The present data reinforce the idea that a control group is needed to ensure that no changes in amplitude have occurred between sessions, as suggested by other authors [20], [21].

A possible explanation for the increment in P3 amplitude could be that the task becomes less difficult in the second session [22], [23], [24]. The behavioral results do not indicate that the task is necessarily easier in the second session, but it is also possible that the behavioral responses have reached a limit. If so, participants cannot improve their behavioral responses but can involve fewer resources in performing the task.

### P3 topography (group analysis)

In the analysis of topographical reliability among groups, the main result was the high correlation values of voltage along the 58 scalp derivations between the grand averages for sessions 1 and 2 (r = 0.977). This score supports the general consensus [3], [7] that the topographical distribution of the P3 component shows really high reliability between sessions.

Regarding the possible modulations in amplitude for specific electrodes, an ANOVA was performed to analyze the possible changes between sessions. The results showed clearly that some derivations were statistically significant especially those surrounding the Pz electrode. To confirm whether the changes in amplitude were caused by changes in the topographical distribution over the scalp, a specific correlation analysis for the electrodes that exhibited statistical differences in the ANOVA showed a high score (0.984), suggesting an increase in amplitude with no significant changes in topography. We propose that this simple method could be an easy way to disentangle amplitude from topography modulations, although more powerful methods have been developed [25]. It is important to emphasize that the topography of the P3 component showed this high stability even when the latencies for exporting the voltage amplitudes were not exactly the same among sessions (see above for a specific comment about this issue).

### P3 Topography (Individual Analysis, Within-Subjects)

In the analysis of topographical stability at individual level, the correlations between sessions were generally very high (ICC mean: 0.888). In some cases the value of 0.981 was reached although there was also a case with a correlation value of 0.626. Some studies [8] have based their conclusions on the proposal of Helmstadter [26]. This author indicated that a correlation of 0.5 or above can be considered acceptable for group studies and 0.94 for individual studies. Under this premise, the stability of the topography scores for the cognitive test employed in this study at group level is more than enough (r = 0.977). However, for the individual values, not all the correlation scores reached the 0.94 level (although some did, see table S1). Nevertheless, some authors have pointed out that in the ERP field a correlation value over 0.54 can be considered enough to guarantee stability of the measure [27]. Granted this last assumption (0.54 is sufficient) and reviewing figure 3 (maps) and the correlation scores described in table S1, the main conclusion is that the topography of the P3 component exhibited a good average level (0.888), so it is possible to check alterations in the topography. No subject in the sample collected showed a correlation value below 0.54. The importance of an individual high reliability level is that it allows us to seek alterations in the topographic parameter. A change in the topographic profile for a subject could be related to pathological processes, as has been described by some authors [19], [25]. However, further studies are necessary to confirm that topography changes could be used as indicators of pathological alteration in brain activity.

### P3 Topography (Individual Analysis, Between-Subjects)

As figure 3 illustrates, each subject has a specific P3 topography. Besides, in almost all cases in session 1, the within-subject correlation was higher than that observed in the between-subject comparisons. These data corroborate what has been described by other authors [9]: each subject responds specifically to the visual stimulation. Some other studies are compatible with this assumption and have indicated natural variability even in the definition of the cerebral structures at the most basic sensory levels [28], [29]. This result is especially relevant since it invites individualized studies of patients according to the evolution of their own P3 topographies. Use of these topographical maps will help to assess the potential benefits of rehabilitation programs or drug therapies in human cognition.

At the same time, a certain common feature was shared by almost every subject from our sample: a maximum amplitude in posterior areas (parietal), in some cases lateralized to the right side of the scalp. The correlation for the group comparison resulted in a really high value (0.977), demonstrating that the factors common to all subjects are highly reproducible. This result is also consistent with studies that have determined genetic factors critical for the P3 component [10], [30].

But genetic factors are not alone in being able to induce a certain homology between topographies for this component. The correlation values among subjects in session 2 reveal an interesting fact: the correlation among different participants was higher than that in session 1. This could be interpreted as trend towards homogenization of the P3 topography based on the automation of information processing, which can reduce the diversity of cognitive mechanisms involved in the task for different subjects. Nevertheless, this reasoning has to be demonstrated by specific experiments in which the task does not become automated.

### Clinical application

Owing to the range of possible P3 topographies observed in the present study, it seems difficult to define a pathological P3 topography for a particular patient. Alternatively, it is possible to consider a group of patients and check differences from the general topography obtained from a control group. However, it must be recalled that this global view hides individual differences within the patient cohort. Considering the high reliability of individual topographies, it would be possible to determine the initial status of a patient and determine how they evolve during treatment using its own topographical profile. This procedure could be used to check if new topographies appear after recovery and would indicate new areas involved in compensation. This will help us to understand the rehabilitation process and how we can stimulate it.

A critical step in obtaining stable P3 parameters is to apply a simple cognitive task that does not involve multiple cognitive mechanisms, as other authors have suggested [7], [31], [32]. In this sense, and reviewing the literature about the stability of P3 parameters, diverse studies have obtained high correlation scores, possibly because the experimental design limited the cognitive mechanisms involved [2], [3], [5], [9], [12]. In other cases, the stability could be affected by the necessary complexity of the task [19], [27] or simply because no specific cognitive task was required (eyes open) [31], [32]. The possible range of neural groups involved in these cases would reduce the reliability of latency, amplitude and topography values. One way to guarantee that the experimental design is simple and to preclude the involvement of multiple neural groups in different trials is to check that there were no differences among sessions in the behavioural responses to the same stimulus. In the present study this condition was satisfied, and this probably explains the high correlation values successfully obtained for all P3 parameters. As a consequence, in defining cognitive tasks to assess their stability, it is important to pay attention to such different issues as the cognitive load, stimulus-response combinations, cognitive strategies and so on.

Finally, some requirements for the long latency ERPs in clinical practice proposed by different authors [21], [33] deserve comment. As these authors have pointed out, these ERPs have to fulfil certain criteria, as other potentials do (i.e. brainstem ERPs), for valid application in the clinical context. A particular criticism is the lack of precision in the peaks of diverse components (i.e. P3). The great difference between the two types of potential (brainstem and P3) is that the number of a priori cognitive mechanisms involved is difficult to compare. The potential number of structures implicit in the formation of the P3 component does not help to concentrate its latency in narrow intervals. However, despite this supposed variability, the component always appears within an approximately 150 ms range (300–450 ms) and it is easily identifiable. Indeed, the high reliability score for this component in the present study was obtained even when the latency of the component was not the same among sessions. Therefore, the peak of the P3 component seems to represent more a general stage in information processing than a specific cognitive mechanism. However, this general stage involving different neural groups yields highly stable parameters and more than acceptable values for their coefficients of variation. As pointed out by Nuwer et al. [34], clinical uses of cognitive potential topographic analyses are still in their infancy. However, as seen in the present study, when the appropriate cognitive test is used, the reliability of the topographical parameter is highly reproducible and offers a precise and objective way for analyzing brain activity related to cognition.

## Conclusion

The present study has allowed us to check that behavioural measures such as P3 parameters (latency and amplitude) in this visual oddball paradigm show high correlations among sessions separated in time. However, it is important to note that the amplitude of the component can increase from session to session for diverse electrodes (with no associated topography change); this should be controlled in longitudinal studies, e.g. of the time course of a pharmacological treatment or a rehabilitation program. With respect to retest reliability for individual P3 topography, the results demonstrate a high correlation in the within-subject comparisons, which allows us to use P3 topography as a tracer of possible changes in follow-up studies not only for groups but also for individuals. Moreover, the P3 scalp distribution in a first comparison is specific for every individual, evidenced by lower correlations in between-subject than within-subject comparisons. This result supports the view that the visual response of the human brain is very specific. However, repetition of exposure to the task homogenizes the P3 maps, suggesting the experience exerts a relevant modulation effect. Both results suggest interesting applications of the topography parameter in the clinical context and for basic studies aimed at disentangling the heterogeneity of brain activity in humans.

## Supporting Information

Table S1 **Correlation values for the topographical maps (within-subject and between-subject).** Within-subject correlations are represented by ICC (Intraclass Correlation, left column). Correlation values for the topographical maps between subjects and for both sessions are calculated by the Pearson product-moment. The values over the empty diagonal represent the correlations between subjects in session 1. Below that empty diagonal are displayed the values for session 2. All bold values are significant after Bonferroni correction (p<0.00001). ICC scores were all significant at the 0.001 level.
(DOCX)
