# Supplementary material for: Retest Reliability of Individual P3 Topography Assessed by High Density Electroencephalography
Source: PLoS One. 2013 May 1;8(5):e62523. doi: 10.1371/journal.pone.0062523 (PMC3641033; doi:10.1371/journal.pone.0062523)
Supplement: Table S1 — Correlation values for the topographical maps (within-subject and between-subject). Within-subject correlations are represented by ICC (Intraclass Correlation, left column). Correlation values for the topographical maps between subjects and for both sessions are calculated by the Pearson product-moment. The values over the empty diagonal represent the correlations between subjects in session 1. Below that empty diagonal are displayed the values for session 2. All bold values are significant after Bonferroni correction (p<0.00001). ICC scores were all significant at the 0.001 level. (DOCX) [file pone.0062523.s001.docx]

|  | **Subjects** |  |  |  |  |  |  |  |  |  |  |  |  |  |  |  |  |  |  |  |  |  |  |
| --- | --- | --- | --- | --- | --- | --- | --- | --- | --- | --- | --- | --- | --- | --- | --- | --- | --- | --- | --- | --- | --- | --- | --- |
| **ICC** | **r Pearson** | 1 | 2 | 3 | 4 | 5 | 6 | 7 | 8 | 9 | 10 | 11 | 12 | 13 | 14 | 15 | 16 | 17 | 18 | 19 | 20 | 21 | 22 |
| 0,938 | 1 |  | **0,83** | **0,56** | **0,71** | 0,30 | **0,59** | **0,80** | **0,59** | **0,61** | 0,33 | **0,72** | **0,86** | **0,70** | **0,78** | **0,87** | **0,86** | **0,68** | 0,39 | 0,52 | 0,45 | **0,55** | **0,63** |
| 0,981 | 2 | **0,80** |  | **0,64** | **0,84** | 0,37 | **0,69** | **0,87** | **0,61** | 0,38 | 0,34 | **0,79** | **0,85** | **0,80** | **0,79** | **0,92** | **0,81** | **0,82** | 0,49 | **0,74** | 0,49 | 0,36 | **0,67** |
| 0,934 | 3 | **0,81** | **0,65** |  | 0,49 | 0,24 | **0,79** | **0,79** | 0,40 | 0,24 | 0,44 | **0,89** | **0,72** | **0,82** | **0,77** | **0,73** | 0,47 | 0,36 | **0,82** | 0,21 | **0,75** | 0,36 | **0,89** |
| 0,958 | 4 | **0,70** | **0,83** | 0,41 |  | **0,69** | **0,55** | **0,62** | 0,54 | 0,46 | 0,25 | **0,59** | **0,71** | **0,71** | **0,72** | **0,78** | **0,57** | **0,80** | 0,42 | **0,69** | 0,30 | 0,26 | 0,45 |
| 0,766 | 5 | 0,28 | **0,55** | -0,04 | **0,63** |  | 0,41 | 0,21 | 0,35 | 0,40 | 0,29 | 0,30 | 0,37 | 0,39 | 0,36 | 0,37 | 0,10 | 0,40 | 0,43 | 0,38 | 0,19 | 0,09 | 0,20 |
| 0,923 | 6 | **0,78** | **0,73** | **0,94** | 0,44 | 0,11 |  | **0,80** | 0,51 | 0,26 | 0,46 | **0,88** | **0,78** | **0,80** | **0,73** | **0,71** | 0,51 | 0,38 | **0,88** | 0,40 | **0,72** | 0,34 | **0,85** |
| 0,968 | 7 | **0,82** | **0,73** | **0,81** | **0,63** | 0,15 | **0,79** |  | 0,53 | 0,49 | 0,39 | **0,90** | **0,83** | **0,82** | **0,87** | **0,90** | **0,78** | **0,56** | **0,65** | 0,45 | **0,75** | **0,61** | **0,87** |
| 0,797 | 8 | **0,69** | **0,91** | **0,67** | **0,66** | 0,48 | **0,78** | **0,64** |  | 0,29 | 0,20 | 0,53 | **0,68** | 0,44 | 0,50 | **0,57** | **0,55** | **0,65** | 0,38 | **0,62** | 0,33 | 0,36 | 0,46 |
| 0,781 | 9 | **0,87** | **0,66** | **0,69** | **0,66** | 0,23 | **0,63** | **0,85** | **0,57** |  | 0,21 | 0,33 | 0,41 | 0,28 | **0,57** | **0,55** | 0,54 | 0,29 | 0,15 | 0,08 | 0,40 | **0,75** | 0,34 |
| 0,626 | 10 | **0,85** | **0,87** | **0,82** | **0,71** | 0,31 | **0,83** | **0,78** | **0,89** | **0,75** |  | 0,46 | 0,46 | 0,53 | 0,53 | 0,43 | 0,22 | 0,21 | **0,54** | 0,03 | 0,52 | 0,25 | 0,51 |
| 0,979 | 11 | **0,86** | **0,73** | **0,96** | 0,52 | 0,09 | **0,92** | **0,77** | **0,73** | **0,70** | **0,89** |  | **0,84** | **0,88** | **0,85** | **0,87** | **0,62** | 0,50 | **0,85** | 0,37 | **0,78** | 0,40 | **0,96** |
| 0,954 | 12 | **0,83** | **0,73** | **0,87** | 0,48 | 0,10 | **0,85** | **0,80** | **0,78** | **0,76** | **0,87** | **0,87** |  | **0,83** | **0,83** | **0,85** | **0,75** | **0,64** | **0,64** | **0,58** | **0,56** | 0,43 | **0,78** |
| 0,892 | 13 | **0,77** | **0,76** | **0,81** | **0,55** | 0,23 | **0,82** | **0,72** | **0,78** | **0,63** | **0,84** | **0,84** | **0,77** |  | **0,84** | **0,83** | **0,55** | **0,58** | **0,75** | 0,39 | **0,61** | 0,28 | **0,83** |
| 0,945 | 14 | **0,92** | **0,83** | **0,89** | **0,73** | 0,24 | **0,86** | **0,85** | **0,79** | **0,82** | **0,93** | **0,93** | **0,89** | **0,82** |  | **0,90** | **0,69** | **0,57** | **0,66** | 0,33 | **0,73** | **0,55** | **0,84** |
| 0,942 | 15 | **0,82** | **0,84** | **0,79** | **0,66** | 0,31 | **0,79** | **0,81** | **0,77** | **0,76** | **0,82** | **0,78** | **0,81** | **0,74** | **0,84** |  | **0,82** | **0,70** | **0,59** | 0,53 | **0,63** | 0,49 | **0,79** |
| 0,902 | 16 | **0,80** | **0,86** | **0,54** | **0,86** | 0,51 | **0,57** | **0,80** | **0,71** | **0,80** | **0,76** | **0,57** | **0,64** | **0,61** | **0,77** | **0,78** |  | **0,61** | 0,24 | **0,56** | 0,38 | **0,56** | **0,55** |
| 0,865 | 17 | **0,90** | **0,92** | **0,80** | **0,78** | 0,35 | **0,78** | **0,83** | **0,83** | **0,80** | **0,91** | **0,84** | **0,84** | **0,80** | **0,93** | **0,88** | **0,87** |  | 0,22 | **0,73** | 0,21 | 0,16 | 0,34 |
| 0,977 | 18 | 0,46 | 0,42 | **0,79** | 0,12 | -0,11 | **0,80** | 0,40 | **0,60** | 0,29 | **0,65** | **0,79** | **0,66** | **0,66** | **0,63** | 0,50 | 0,12 | 0,49 |  | 0,14 | **0,76** | 0,22 | **0,86** |
| 0,865 | 19 | 0,20 | **0,55** | -0,11 | **0,58** | **0,80** | 0,05 | 0,20 | 0,44 | 0,24 | 0,23 | -0,08 | 0,04 | 0,18 | 0,14 | 0,33 | **0,61** | 0,34 | -0,28 |  | 0,00 | 0,04 | 0,18 |
| 0,889 | 20 | **0,67** | 0,43 | **0,89** | 0,24 | -0,30 | **0,80** | **0,68** | 0,50 | **0,59** | **0,72** | **0,88** | **0,79** | **0,69** | **0,78** | **0,58** | 0,31 | **0,62** | **0,81** | -0,40 |  | **0,55** | **0,86** |
| 0,683 | 21 | **0,74** | 0,49 | **0,93** | 0,35 | -0,18 | **0,84** | **0,83** | 0,54 | **0,75** | **0,75** | **0,87** | **0,83** | **0,71** | **0,84** | **0,67** | 0,50 | **0,70** | **0,69** | -0,22 | **0,91** |  | 0,47 |
| 0,973 | 22 | **0,81** | **0,70** | **0,97** | 0,46 | -0,02 | **0,93** | **0,80** | **0,73** | **0,68** | **0,88** | **0,98** | **0,89** | **0,83** | **0,91** | **0,77** | **0,56** | **0,82** | **0,80** | -0,12 | **0,91** | **0,91** |  |
